# Case of Hydatid Ascites

**Published:** 1825-08

**Authors:** R. Long

**Affiliations:** Surgeon to the Arthurstown Dispensary.


ART. VII.—*Case of Hydatid Ascites.* By R. LONG, M.D. Surgeon to the Arthurstown Dispensary.

MARGARET DALY, æt. thirteen years, the child of parents in extreme poverty, after an imperfect recovery from typhous fever, was seized with a constant, and sometimes very acute, pain in the region of the umbilicus. Her appetite was voracious, bowels irregular, countenance squalid, and the whole body much emaciated. She was placed under an alterative mercurial course. Oil of turpentine emulsion was occasionally given, and a nutritious diet procured.

The abdomen, however, rapidly enlarged, and fluctuation was soon perceptible: tapping of the belly became, therefore, quickly necessary, and five quarts of fluid, nearly colourless, were drawn off. Notwithstanding the most persevering use of purgatives, diuretics and mercurials, paracentesis was performed five times, at intervals of three weeks only. The patient's strength was, however, greatly improved, the appetite good, and the emaciated appearance of the extremities much altered for the better.

After the last operation, a tumor formed at the umbilicus, resembling exompholos, soft, and easily reducible on pressure. As the abdomen became again enlarged, this tumor increased to an enormous size; the integuments became thin and pellucid; and, when tapping was again required, I determined on performing the operation here, it being then evident the tumor was formed by the dropsical contents of the abdomen. The first stroke of the lancet was followed by an immense rush of water, and subsequently the protrusion of several transparent vesicles, which, as they gradually emerged from the aperture, became distended with water also. The chain of vesicles thus formed, and suspended by a pedicle thick as the little finger, soon reached from the umbilicus to the floor on which the chair was placed in which the patient sat, presenting a resemblance to an immense bunch of grapes. I divided the pedicle at the umbilicus, and removed the entire mass; which at length convinced the bystanders that the child's intestines were not come out.

The wound, which was simply dressed with oiled lint, soon healed; the umbilicus resumed its natural appearance; all pain and uneasiness vanished; and, up to this period (now twelve months), there has not been the slightest return of dropsical disease, or other deviation from perfect health.

***Aldertown House, Arthurstown; 20th June, 1825.***

